# Prognostic Factors in Childhood Anaplastic Large Cell Lymphoma: Long Term Results of the International ALCL99 Trial

**DOI:** 10.3390/cancers12102747

**Published:** 2020-09-24

**Authors:** Lara Mussolin, Marié-Cecilé Le Deley, Elisa Carraro, Christine Damm-Welk, Andishe Attarbaschi, Denise Williams, Amos Burke, Keizo Horibe, Atsuko Nakazawa, Grazyna Wrobel, Georg Mann, Monika Csóka, Anne Uyttebroeck, Rafael Fernández-Delgado Cerdá, Auke Beishuizen, Karin Mellgren, Birgit Burkhardt, Wolfram Klapper, Suzanne D. Turner, Emanuele S.G. d’Amore, Laurence Lamant, Alfred Reiter, Wilhelm Woessmann, Laurence Brugières, Marta Pillon, on behalf of the European Inter-Group for Childhood Non-Hodgkin lymphoma (EICNHL)

**Affiliations:** 1 Maternal and Child Health Department, Padua University, 35128 Padua, Italy; elisa.carraro87@gmail.com; 2 Istituto di Ricerca Pediatrica Città della Speranza, 35127 Padua, Italy; 3 Centre Oscar Lambret, Department of Biostatistics, CEDEX 59020 Lille, France; m-ledeley@o-lambret.fr; 4 University Medical Center Hospital Hamburg-Eppendorf, Pediatric Hematology and Oncology, 20246 Hamburg, Germany; c.damm-welk@uke.de (C.D.-W.); w.woessmann@uke.de (W.W.); 5 Department of Pediatric Hematology and Oncology, St. Anna Children’s Hospital, Medical University of Vienna, 1090 Vienna, Austria; andishe.attarbaschi@stanna.at (A.A.); georg.mann@stanna.at (G.M.); 6 Department of Paediatric Haematology, Oncology and Palliative Care, Cambridge University Hospitals NHS Foundation Trust, Addenbrooke’s Hospital, Cambridge CB2 0QQ, UK; denise.williams@addenbrookes.nhs.uk (D.W.); amos.burke@addenbrookes.nhs.uk (A.B.); 7 Clinical Research Centre, National Hospital Organization Nagoya Medical Center, Nagoya 460-0001, Japan; keizo.horibe@nnh.go.jp; 8 Department of Clinical Research, Saitama Children’s Medical Center, Saitama 330-8777, Japan; nakazawa.atsuko@scmc.pref.saitama.jp; 9 Department of Bone Marrow Transplantation, Children Oncology and Hematology, Wroclaw Medical University, 50-367 Wroclaw, Poland; wrobel.wroc@wp.pl; 10 2nd Department of Pediatrics, Semmelweis University, 1094 Budapest, Hungary; csoka.monika@med.semmelweis-univ.hu; 11 Department of Pediatric Hematology and Oncology, University Hospitals Leuven, 3000 Leuven, Belgium; anne.uyttebroeck@uzleuven.be; 12 Department of Pediatrics, Obstetrics and Gynaecology, University of Valencia, 46010 Valencia, Spain; rafael.fdez-delgado@uv.es; 13 Princess Máxima Center for Pediatric Oncology, 3584 CS Utrecht, The Netherlands; a.beishuizen-2@prinsesmaximacentrum.nl; 14 Department of Pediatric Haematology and Oncology, Sahlgrenska University Hospital, 41685 Gothenburg, Sweden; karin.mellgren@vgregion.se; 15 Department of Pediatric Hematology and Oncology, University Hospital Muenster, D-48149 Muenster, Germany; birgit.burkhardt@ukmuenster.de; 16 Institute of Pathology, Hematopathology, University Hospital Schleswig Holstein, D-24105 Kiel, Germany; wklapper@path.uni-kiel.de; 17 Department of Pathology, University of Cambridge, Cambridge CB2 1QP, UK; sdt36@cam.ac.uk; 18 Central European Institute of Technology (CEITEC), Masaryk University, 60200 Brno, Czech Republic; 19 Department of Pathological Anatomy, San Bortolo Hospital, 36100 Vicenza, Italy; dir.anatpat@ulssvicenza.it; 20 Department of Pathology, Institut Universitaire du Cancer Toulouse Oncopole, France—Université Toulouse III-Paul Sabatier, UMR1037 CRCT, F-31000 Toulouse, France; lamant.l@chu-toulouse.fr; 21 Department of Pediatric Hematology and Oncology, Justus Liebig-University Giessen, 35390 Giessen, Germany; alfred.reiter@paediat.med.uni-giessen.de; 22 Department of Pediatrics and Adolescents Oncology, Gustave Roussy, 94800 Villejuif, France; laurence.brugieres@gustaveroussy.fr; 23 Pediatric Hematology, Oncology and Stem Cell Transplant Division, Padua University Hospital, 35128 Padua, Italy; marta.pillon@unipd.it

**Keywords:** ALCL, childhood, MDD, long-term follow-up

## Abstract

**Simple Summary:**

The long-term follow-up and the results of the analysis of clinical, pathological and molecular prognostic factors for 420 children, adolescents and young adults affected by anaplastic large cell lymphoma (ALCL), enrolled in the international ALCL99 trial, were reported. The 10-year follow-up highlighted a progression free survival of 70% and an overall survival of 90%; rare late relapses occurred and no secondary malignancies were registered. Among clinical and pathological characteristics, only morphology including the small cell/lymphohistiocytic (SC/LH) pattern was independently associated with the risk of disease progression or relapse. When available minimal disseminated disease (MDD) data (*n* = 162) were included, both SC/LH pattern and MDD positivity resulted significantly associated with a poorer outcome when assessed by multivariate analysis. Considering MDD and SC/LH results, three biological/pathological risk groups with significantly different prognoses were identified. These results should be considered in the design of future ALCL trials to tailor individual treatments.

**Abstract:**

With the aim of describing the long-term follow-up and to define the prognostic role of the clinical/pathological/molecular characteristics at diagnosis for childhood, adolescent and young adults affected by anaplastic large cell lymphoma (ALCL), we analyzed 420 patients aged up to 22 years homogeneously treated within the international ALCL99 trial. The 10-year progression free survival (PFS) was 70% and overall survival was 90%, rare late relapses occurred but no secondary malignancies were reported. Among clinical/pathological characteristics, only patients presenting a small cell/lymphohistiocytic (SC/LH) pattern were independently associated with risk of failure (hazard ratio = 2.49). Analysis of minimal disseminated disease (MDD), available for 162 patients, showed that both SC/LH pattern (hazard ratio = 2.4) and MDD positivity (hazard ratio = 2.15) were significantly associated with risk of failure in multivariate analysis. Considering MDD and SC/LH results, patients were separated into three biological/pathological (bp) risk groups: a high-risk group (bpHR) including MDD-positive patients with SC/LH pattern; a low-risk group (bpLR) including MDD-negative patients without SC/LH pattern; and an intermediate-risk group (bpIR) including remaining patients. The 10-year PFS was 40%, 75% and 86% for bpHR, bpIR and bpLR, respectively (*p* < 0.0001). These results should be considered in the design of future ALCL trials to tailor individual treatments.

## 1. Introduction

Anaplastic large cell lymphoma (ALCL) accounts for 15% of pediatric and adolescent non-Hodgkin lymphomas (NHLs) [1]. ALCL in children is nearly universally positive for Anaplastic Lymphoma Kinase (ALK) and in almost all cases is characterized by the t(2;5)(p23;q35) translocation, resulting in expression of the hybrid oncogenic tyrosine kinase *NPM-ALK* [2].

Mediastinal involvement, visceral involvement defined as lung, liver or spleen involvement and skin lesions have previously been identified as poor prognostic factors [3]. In addition, progression free survival (PFS) and overall survival (OS) were significantly lower for patients with at least one clinical risk factor (61% and 73%, respectively) compared to patients without risk factors (89% and 94%, respectively) [3]. These factors were used for patient stratification in the first randomized ALCL clinical trial of the European Inter-Group for Childhood Non-Hodgkin lymphoma (EICNHL), the ALCL99 trial [4,5]. The results of survival analysis at 2 years showed the event free survival (EFS) to range from 70% to 75%, without a significant difference between the randomized arms [4,5]. So far, data regarding survival of children with ALCL have only been reported at 2 or 5 years [3,4,5,6,7,8,9,10,11] with long-term follow-up never previously reported. Long-term follow-up data for ALCL patients after standard chemotherapy is needed for the design of new therapeutic trials and to judge the risk of late relapse.

Several biological and pathologic characteristics have been shown to be associated with a higher risk of treatment failure in childhood ALK-positive ALCL over the past 15 years, i.e., high risk morphological pattern by the presence of a small cell (SC) or lymphohistiocytic (LH) pattern [12], positive PCR for *NPM-ALK* in peripheral blood (PB) and/or bone marrow (BM) at diagnosis (minimal disseminated disease; MDD) [13,14] and low anti-ALK antibody titers at diagnosis [15,16]. However, all these analyses were based on different cohorts, or evaluated by one or two collaborative international groups.

The aim of the present study was to describe the long-term, 10-year follow-up of children, adolescents and young adults (CAYA) with ALCL and to confirm a prognostic role for the clinical, pathological and molecular characteristics available at diagnosis of the large group of patients treated within the ALCL99 protocol for systemic ALCL, and to better define risk stratification.

## 2. Methods

### 2.1. Patients and Clinical-Pathological Factors

Between November 1999 (start of the study) and June 2006 (end of accrual in the randomized trial), 529 patients aged <22 years with a diagnosis of ALCL were registered to the ALCL99 trial, which was conducted by 10 national or cooperative groups including European and Japanese pediatric/lymphoma study groups (NCI NCT00006455). The study was approved by local ethics committees and informed consent was obtained from all patients/guardians.

The diagnosis of ALCL was based on morphologic and immunophenotypic characteristics including CD30 and ALK immunostaining and, whenever possible, by molecular definition (evidence of ALK fusion genes for ALK + ALCL). Each national histologic diagnosis was reviewed by an international panel of 10 hematopathologists to confirm diagnosis and to define a consensus on morphological pattern ensuring high quality of the final classification [12].

Patients were staged according to the Ann Arbor and St Jude staging systems [17,18]. Patients were classified into clinical risk groups as high-risk (HR) if they presented with skin, mediastinal or visceral involvement (defined as lung, liver or spleen involvement), or as standard-risk (SR) if they had none of the previous sites of disease, or as low-risk (LR) if they were in stage I with completely resected disease. Patients with isolated skin lesions (most often CD30-positive cutaneous lymphoproliferations) or with CNS involvement were not eligible for the randomized study but registered in the database. Patients with CNS involvement were recently described [19]. Treatment and response criteria for all eligible patients were conducted according to the ALCL99 protocol (Table S1) [4,5].

### 2.2. Minimal Disseminated Disease (MDD)

MDD was measured by qualitative PCR for *NPM-ALK* in BM or PB as previously described [16]. A patient was considered MDD-positive if at least one of the two samples (BM or PB) was PCR-positive. Quality control was performed between EICNHL central laboratories demonstrating identical results with serially diluted *NPM-ALK*-positive cell lines and blinded patient samples [16]. MDD analysis was not mandatory and was only performed in a few select laboratories of the participating countries.

### 2.3. Statistical Analysis

The first prognostic factor analysis was performed for all patients included in the database except those with isolated skin lesions or with CNS involvement (who were registered but received different treatments). Variables included in the analysis of prognostic factors were (i) clinical characteristics (age, sex, B-symptoms, lymph node, mediastinal involvement, skin lesions, bone and BM involvement, soft tissue mass, visceral, spleen, liver and lung involvement and lactate dehydrogenase (LDH) serum level); (ii) pathological data including ALK immunostaining, CD3 expression and morphological patterns classified into two categories: ALCL with or without an SC or LH pattern (defined as the presence of either SC or LH morphological patterns whether or not these were associated with another morphological pattern); (iii) staging system: St Jude classification, Ann Arbor staging, ALCL99 prognostic groups and age-adjusted International Prognostic Index (IPI). Then, for patients with retrospectively collected MDD data available, additional prognostic analysis including MDD data together with the above described characteristics was performed.

Associations between patient characteristics were analyzed with Chi-squared or Fisher’s exact tests. The main endpoint for the prognostic factor study was the PFS, defined as the time elapsed between the date of biopsy and the date of occurrence of disease progression or relapse. Patients alive without treatment failure were censored at the date of last follow-up, which was collected in July 2018. Patients who died without previous disease progression or relapse were censored at the date of death. The OS was also assessed, defined as the time elapsed between the date of biopsy and the date of death, whatever the cause. All survival probabilities were estimated using the Kaplan–Meier method [20]. Cox model was performed to examine the risk factors affecting the PFS in the multivariate analyses, including variable with *p* < 0.2 in univariate analysis [21]. All *p*-values were two-sided, with a type I error rate fixed at 0.05. Statistical analysis was carried out using the SAS statistical program (SAS-PC, version 9.4; SAS Institute Inc., Cary, NC, USA).

## 3. Results

### 3.1. Patient Characteristics

**Figure 1:**
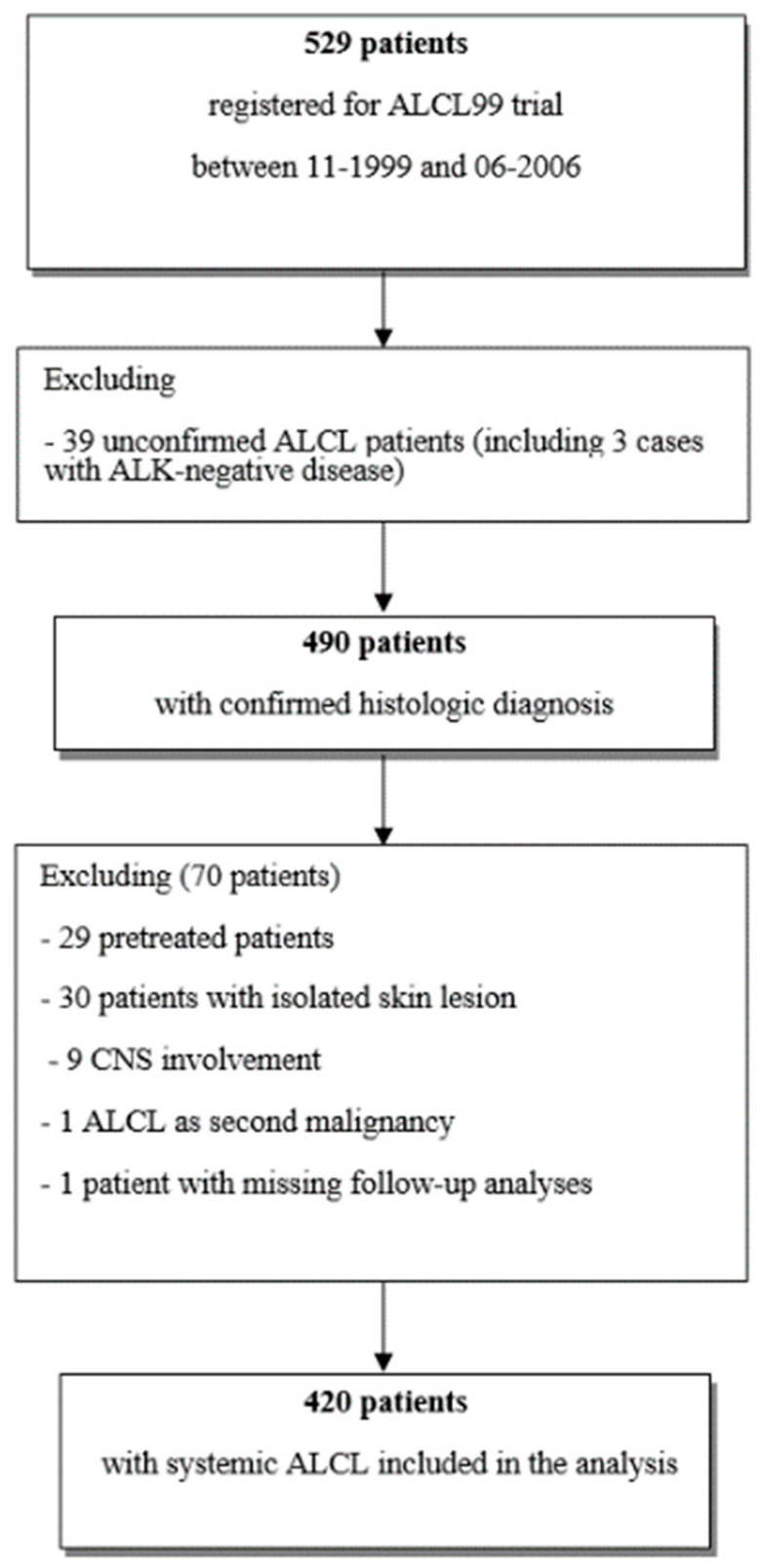
Flow chart of anaplastic large cell lymphoma (ALCL) patients included in the analysis.

Of 529 patients registered in the database, 109 were excluded. Thus, 420 patients were evaluable for the current analysis (Figure 1).

Among the 420 patients, 256 were males and 164 females (male-to-female ratio, 1.6:1); the median age at diagnosis was 11 years (range, 0.8–19.5 years). Overall, 370 patients were randomized into the Methotrexate (MTX)-arm of the trial and 205 into the Vinblastine (VLB)-arm. The histological diagnosis was based on international panel review or national review of 95% and 5% of patients, respectively. The clinical and pathological characteristics of the patients are depicted in Table 1. The median follow-up for 420 patients was 9.2 years (range, 0.03–17.9 years).

Overall, 125 patients experienced disease progression or relapse, and 90 of them (72%) underwent a second-line (or further) treatment achieving complete remission (CR). The remaining 35 patients (38%) never achieved CR and died of tumor progression (*n* = 24) or due to treatment related mortality (TRM) (*n* = 11). Six other patients died of TRM in their first CR during treatment. Overall 41 patients died, 35 after a disease progression or relapse. No secondary malignancies were observed during the period of long-term follow-up.

**Figure 2:**
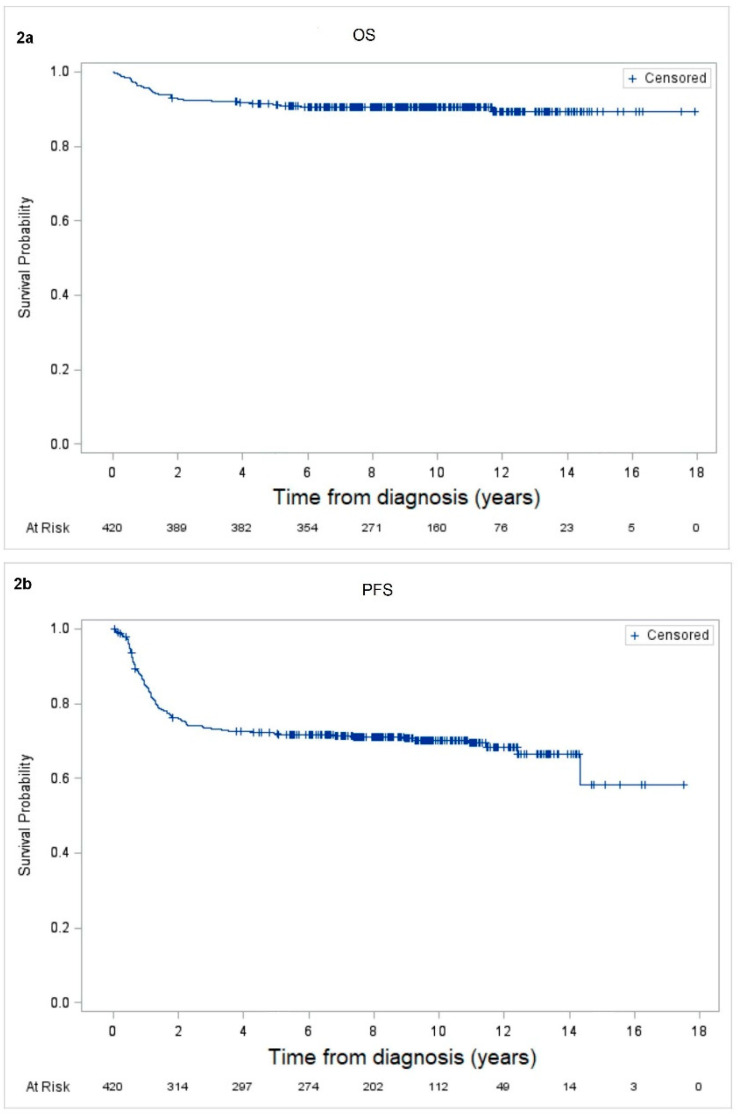
The 10-year overall survival (OS) and progression free survival (PFS) were 90% (SE ± 1%) (**a**) and 70% (SE ± 2%) (**b**), respectively, for the full cohort of 420 patients.

The OS was 91% (SE ± 1%) and 90% (SE ± 1%) at 5 and 10 years, respectively. The PFS was 72% (SE ± 2%) and 70% (SE ± 3%) at 5 and 10 years, respectively (Figure 2).

### 3.2. Analysis of Prognostic Factors for Risk of Disease Progression or Relapse for the 420 Eligible Patients

Table 1 details the results of univariate and multivariate analyses of risk of disease progression or relapse, considering clinical and pathological characteristics. As was previously demonstrated, the type of treatment allocated by randomization did not impact patient outcome; this was not taken into account in the main analysis. In univariate analysis, mediastinal involvement (*p* = 0.03), peripheral lymph node involvement (*p* = 0.02) and the presence of a SC/LH pattern (*p* < 0.0001) at diagnosis were all associated with a worse prognosis (Table 1). These three variables, together with other variables that have a *p* < 0.20 as determined by univariate analysis (St. Jude staging system, defined as stage I + II vs. III + IV, Ann Arbor staging system, defined as 1 + 2 vs. 3 + 4, risk group, and B-symptoms) were entered into multivariate analysis. Only SC/LH morphological pattern retained an independent association with the risk of disease progression or relapse (hazard ratio = 2.49, *p* < 0.0001) (Table 1).

**Figure 3:**
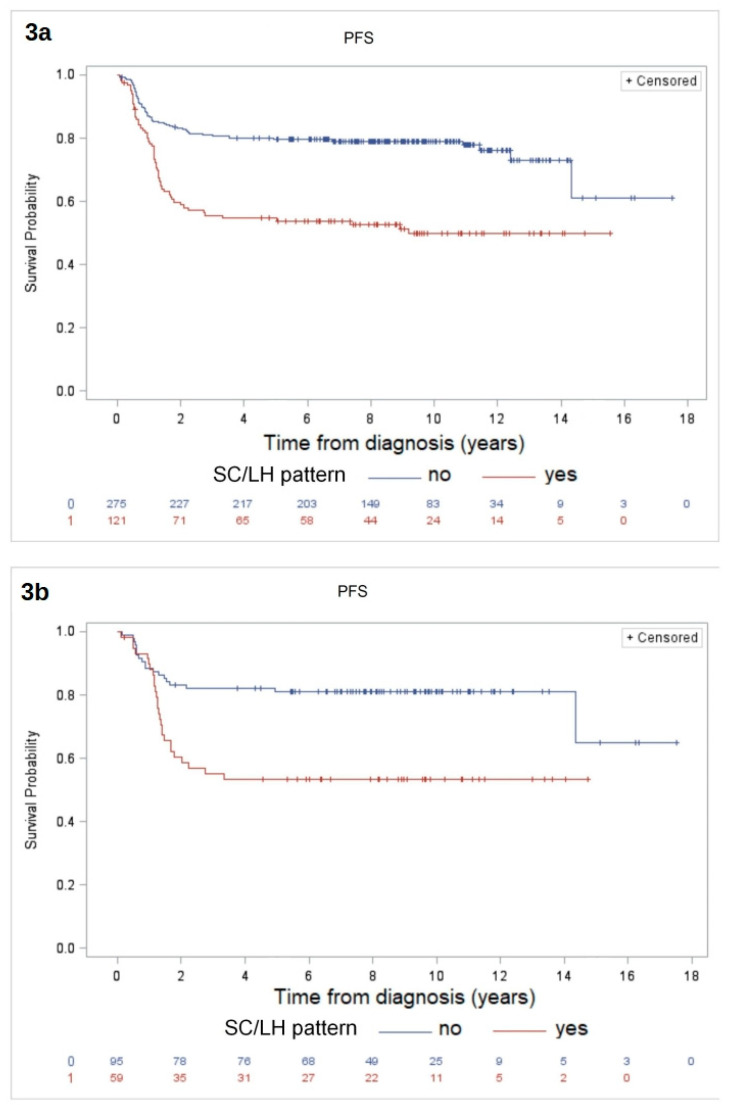
The 10-year PFS for the full cohort of 420 patients was 50% (SE ± 5%) in the SC/LH subtype group and 78% (SE ± 2%) in the non-SC/LH group, *p* < 0.0001, (**a**); the 10-year PFS in the subset of 162 patients with available MDD data was 53% (SE ± 7%) in SC/LH group and 81% (SE ± 4%) in the non-SC/LH group, *p* = 0.0006 (**b**).

In particular, the 10-year PFS was 50% (SE ± 5%) for patients with an SC/LH pattern as compared to 79% (SE ± 2%) for other morphologies, *p* < 0.0001 (Figure 3a). Analysis of the statistical associations among clinical and pathological prognostic factors for the 420 eligible patients are reported in Appendix A.

An additional analysis was performed for the subgroup of patients with an age-adjusted IPI score and CD3 expression data available. The age-adjusted IPI score and CD3 immunostaining data were available for 329/420 (78%) and 322/420 (77%) patients, respectively. Patients stratified for IPI score showed a 10-year PFS of 74% (SE ± 3%) and 59% (SE ± 6%) for IPI 0–2 (*n* = 263) and IPI 3 (*n* = 66), respectively (*p* = 0.02). Analysis of CD3 expression at diagnosis showed a 10-year PFS of 74% (SE ± 3%) for CD3-negative patients (*n* = 268) and 56% (SE ± 7%) for CD3-positive patients (*n* = 54), *p* = 0.04. The multivariate analysis, performed in the subgroup of patients for whom IPI score and CD3 data were available (*n* = 232), confirmed the negative prognostic value of an SC/LH pattern (Table S2).

### 3.3. Analysis of Prognostic Factors for Risk of Disease Progression or Relapse Taking-into-Account MDD Analysis

MDD analysis was conducted for a total of 162 systemic *NPM-ALK*-positive ALCL patients recruited to the ALCL99 trial for whom BM and/or PB samples were available. Qualitative PCR was performed by four national groups. Among the 162 patients with BM samples available, 100 had matched PB samples. Concordance between BM and PB was high, showing the same results in 82/100 cases (82%), whereas the test was positive in PB and negative in BM for 11 cases, and negative in PB and positive in BM for 7 cases. Clinical, pathological and molecular characteristics of this subset of patients are reported in Table 2. The distribution of the clinical and pathological characteristics was similar to the distribution of the whole cohort of 420 patients. Among the 162 patients with available MDD data, 87 (54%) were MDD-positive and 75 (46%) were MDD-negative. The median follow-up of the 162 patients was 9 years (range, 0.03 to 18 years). During follow-up, 46 treatment failures and 13 deaths were observed. The 10-year PFS for the 162 patients was 72% (SE ± 4%) and the 10-year OS was 92% (SE ± 2%). Reported characteristics were assessed by univariate and multivariate analyses (Table 2). By univariate analysis, only the SC/LH pattern (*p* = 0.0006) and MDD-positivity (*p* = 0.001) significantly associated with a higher risk of disease progression or relapse. The multivariate model included the St. Jude classification (I + II vs. III + IV), Ann Arbor staging system (1 + 2 vs. 3 + 4), mediastinal involvement, soft tissue mass, peripheral lymph node, BM infiltration, CD3 positivity, SC/LH pattern and MDD as they had a *p*-value of less than 0.20 as determined by univariate analysis. Both SC/LH morphological pattern and MDD positivity were significantly associated with the risk of failure as assessed by multivariate analysis, with a hazard ratio = 2.4, *p* = 0.009, and a hazard ratio = 2.15, *p* = 0.038, respectively.

**Figure 4:**
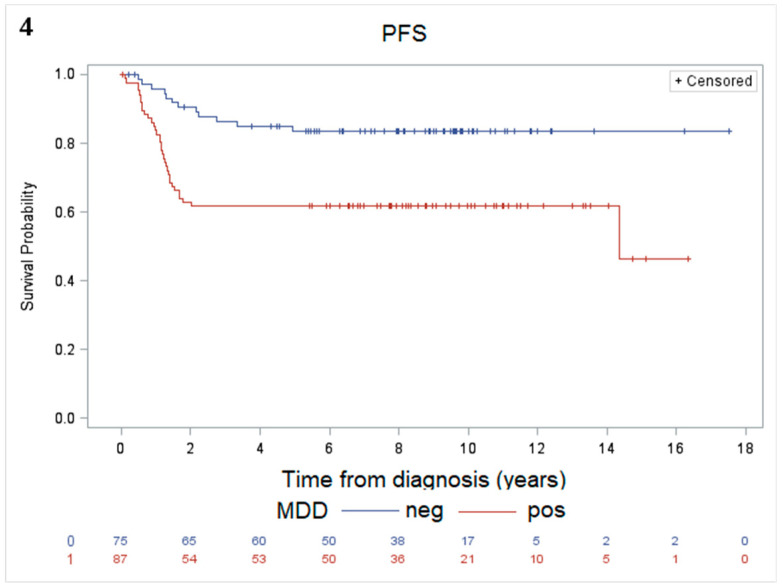
The 10-year PFS according to MDD stratification was 62% (SE ± 5%) for the MDD positive patients and 83% (SE ± 4%) for the MDD negative patients, *p* = 0.001.

The 10-year PFS was 53% (SE ± 7%) in the SC/LH group and 81% (SE ± 4%) in the non-SC/LH group, *p* = 0.0006 (Figure 3b). The 10-year PFS according to MDD stratification was 62% (SE ± 5%) for the MDD positive group and 83% (SE ± 4%) for the MDD negative group, *p* = 0.001 (Figure 4). Analysis of the statistical associations among clinical, pathological and biological prognostic factors for the 162 patients with MDD evaluation are reported in Appendix A.

**Figure 5:**
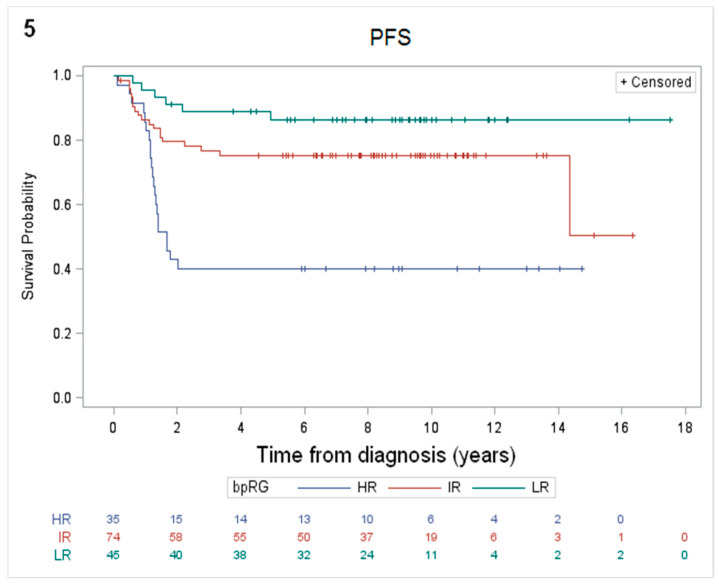
The 10-year PFS was 40% (SE ± 8%), 75% (SE ± 5%) and 86% (SE ± 5%) for bpHR (*n* = 35), bpIR (*n* = 74) and bpLR (*n* = 45), respectively (*p* < 0.0001).

In addition, in an attempt to highlight different biological/pathological (bp) risk groups, a combination of the two independent prognostic factors identified by multivariate analysis (MDD and SC/LH pattern) was tested. Thus, patients were stratified into 3 new risk groups: (1) a high-risk group (bpHR) including MDD-positive patients and SC/LH morphological pattern (35/154 patients, 23%); (2) a low-risk group (bpLR) including MDD-negative patients without SC/LH pattern (45/154 patients, 29%); (3) an intermediate-risk group (bpIR) including all other patients with one of the two factors (74/154 patients, 48%). Univariate analysis showed a 10-year PFS of 40% (SE ± 8%), 75% (SE ± 5%) and 86% (SE ± 5%) for bpHR (*n* = 35), bpIR (*n* = 74) and bpLR (*n* = 45), respectively (*p* < 0.0001) (Figure 5). The 10-year OS analysis according to the bp risk groups did not show any statistical differences among the 3 groups (Figure S1).

## 4. Discussion

We report the long-term follow-up and explored potential prognostic factors for relapse in a large cohort of 420 children, adolescents and young adults with systemic ALCL treated homogeneously within the international ALCL99 trial across 10 countries between 1999 and 2006. The randomized ALCL99 trial demonstrated that the dose and mode of administration of high dose MTX in the MTX-arm, for SR and HR patients and the addition of VBL to the ALCL99 backbone in the VBL-arm, for HR patients, did not impact the risk of failure [4,5]. These findings provide the unique opportunity to describe and analyze prognostic factors in the largest cohort of prospectively collected data from patients with this rare disease and a very long follow-up of on average 10 years. The 2-year survival analysis previously reported for the MTX-arm (OS of 92.5% and EFS of 74.1%) [4] and for the VBL-arm (OS of 94% and EFS of 71%) [5] were stable over time. Our analysis confirms a risk of relapse of about 30% and shows that ALCL patients had a reasonable chance of rescue at relapse reaching an OS of about 90%. PFS and OS were similar at 5 and 10 years from diagnosis, with very rare events observed after the fifth year. Of note, no secondary malignancies were registered. In the present analysis, patients with HR criteria, as defined in the ALCL99 trial, unexpectedly did not have a higher risk of relapse. Although our analysis confirmed by univariate analysis the negative prognostic value of mediastinal involvement (*p* = 0.03), we have not been able to confirm the negative prognostic value of skin and/or visceral involvement. None of the other clinical characteristics were associated with the risk of relapse as determined by univariate analysis, except for peripheral lymph node involvement (*p* = 0.02), age-adapted IPI score 3 (*p* = 0.02) and CD3 positivity (*p* = 0.04); these last two variables unfortunately could not be tested in the global multivariate analysis due to high number of missing data. It was observed that lymph nodes were involved in almost 90% of patients; the remaining 48 patients without lymph node involvement at diagnosis, and with better prognosis, were patients with rare sites of presentation, mostly soft tissue masses (40%) or bone lesions (33%), independent of the overall stage.

Our analysis has confirmed the negative prognostic value of an SC/LH pattern as previously reported by Lamant in 2011 based on a preliminary histological analysis of ALCL99 trial data [12]. Patients with SC/LH pattern could be hypothesized to have low levels of immune activation against tumor cells since the presence of the SC/LH pattern has been shown to be associated with low anti-ALK antibody titers at initial diagnosis [15]. Even though the presence of an SC/LH pattern has been shown to be associated with a higher risk of failure in several studies [12,14,16], this criterium is not used to stratify treatment as it requires centralized pathology review and further international testing to evaluate its reproducibility. In fact, the concordance between national and international reviews was good but not excellent (κ index equal to 0.67), suggesting that highly reproducible specific criteria have yet to be defined [12]. In addition, identifying biomarkers associated with this subtype might help to implement a more precise definition and tailored treatment.

MDD data were not available for all ALCL patients included in the ALCL99 trial since MDD analysis was not mandatory and was only performed in a few select laboratories of the participating countries. Harmonization and quality control of MDD methods had been introduced among these laboratories facilitating the inclusion of all data into a common analysis. The multivariate analysis performed on the subgroup of 162 ALCL patients with available MDD results, confirmed that both MDD-positivity and the presence of a SC/LH pattern were independently associated with relapse. This allowed for the definition of three risk groups: the bpHR group, including MDD-positive patients with SC/LH pattern, accounting for 29% of patients, had a worse prognosis with a 10-year PFS of 40% compared to the bpLR group, including MDD-negative patients without the SC/LH pattern, with a 10-year PFS of 86%. The bpIR group, including all other patients, showed a 10-year PFS of 75%. The 10-year OS according to the bp risk groups did not show any statistical difference, confirming that the salvage therapies are highly effective for pediatric patients affected by ALCL. Several other risk factors for ALCL such as minimal residual disease (MRD) or anti ALK-antibody titers have previously been reported by other international collaborative groups [16,22,23,24]. The number of patients with available data regarding MRD or ALK-antibodies in the current analysis was too small to allow for analysis including these parameters.

Anyway, the characterization of biological/pathological groups of pediatric patients with poor outcome could accelerate the availability of well-known targeted therapies also for the first line treatment during childhood. In fact, to date, the experience in pediatric age of promising drugs like anti-CD30 antibody-drug conjugate, last-generation ALK-inhibitors and immune check-point inhibitors, is still limited to clinical trials or ad hoc off-label use [24].

## 5. Conclusions

In conclusion, the results described here have allowed us to propose risk stratification parameters for new international trials for the treatment of children, adolescents and young adults affected by ALCL based on MDD results and SC/LH morphological pattern. In particular, in this scenario, high-risk patients who are MDD positive at diagnosis could benefit more from the last generation targeted drugs.

## Figures and Tables

**Table 1:** Patient and disease characteristics of the whole study cohort (420 patients).

Patient Characteristics		# Patients	# Events	10-y PFS % (SE %)	Univariate *p*-Value	Multivariate *p*-Value	Hazard Ratio (95% CI)
Sex	M	256	73	71 (3)	0.38		
	F	164	52	69 (3)			
Age (years)	<11	206	62	70 (3)	0.87		
	≥11	214	63	71 (3)			
LDH (IU/L)	<361	199	58	72 (3)	0.67		
	≥361	221	67	69 (3)			
St. Jude Stage System *	I + II	109	28	74 (4)	0.17	0.87	
	III + IV	311	97	69 (3)			
Ann Arbor Stage System °	1 + 2	166	43	74 (4)	0.1	0.73	
	3 + 4	25	82	68 (3)			
Risk group ^	LR + SR	162	42	75 (4)	0.11	0.15	
	HR	258	83	63 (3)			
Age-adjusted IPI (91 mv)	0–2	263	68	74 (3)	0.02 ^+^		
	3	66	26	59 (6)			
B symptoms (5 mv)	No	185	47	74 (3)	0.08	0.14	
	Yes	230	74	68 (3)			
Mediastinum involvement	No	227	58	75 (3)	0.03	0.14	
	Yes	193	67	65 (4)			
Lung lesions	No	335	98	71 (3)	0.3		
	Yes	85	27	68 (5)			
Liver involvement	No	360	104	71 (2)	0.22		
	Yes	60	21	63 (7)			
Spleen involvement	No	347	101	71 (3)	0.28		
	Yes	73	24	68 (6)			
Skin lesions	No	313	88	72 (3)	0.3		
	Yes	107	37	65 (5)			
Soft tissue mass (27 mv)	No	356	112	68 (3)	0.22		
	Yes	37	8	81 (6)			
ENT involvement (10 mv)	No	405	120	70 (2)	0.61		
	Yes	5	1	80 (18)			
Peripheral LN involvement	No	48	7	88 (5)	0.02	0.08	
	Yes	372	118	68 (2)			
Abdominal LN involvement	No	230	67	71 (3)	0.83		
	Yes	190	58	69 (4)			
Visceral (lung, liver, spleen) involvement	No	284	82	72 (3)	0.29		
	Yes	136	43	68 (4)			
Kidney and/or pancreas lesions	No	394	117	70 (2)	0.73		
	Yes	26	8	69 (9)			
Bone lesions	No	353	108	70 (3)	0.43		
	Yes	67	17	74 (5)			
Bone marrow involvement	No	379	111	70 (2)	0.62		
	Yes	41	14	69 (9)			
ALK immunostaining	Neg	16	4	75 (11)	0.62		
	Pos	404	121	70 (2)			
CD3 immunostaining (98 mv)	Neg	268	73	74 (3)	0.04+		
	Pos	54	23	56 (7)			
Histological subtype SC/LH (24 mv)	No	275	61	79 (2)	<0.0001	<0.0001	2.49 (1.71–3.63)
	Yes	121	58	50 (5)			

Events: progression or relapse; PFS: progression free survival; SE: standard error; M: male; F: female; LDH: lactate dehydrogenase, median value 361 (range 200–9512 IU/L); LR: low risk; SR: standard risk; HR: high risk; IPI: International Prognostic Index; mv: missing value; ENT: ears nose throat; LN: lymph nodes; ALK: anaplastic lymphoma kinase; Neg: negative; Pos: positive; ALK: Anaplastic Lymphoma Kinase; SC/LH: small cell or lymphohistiocytic; * Patients’ number according to St. Jude Stage System: 33 (stage I), 76 (stage II), 270 (stage III), 41 (stage IV); ° Patients’ number according to Ann Arbor Stage System: 38 (stage 1), 128 (stage 2), 139 (stage 3), 125 (stage 4); ^ Patients’ number according to LR and SR Risk group: 6 (LR), 156 (SR). + Patient characteristics not included in multivariate analysis (despite univariate *p*-value < 0.20) due to the high number of missing values.

**Table 2:** Patient and disease characteristics of the 162 patients with minimal disseminated disease data available.

Patient Characteristics		# Patients	# Events	10-y PFS % (SE %)	Univariate *p*-Value	Multivariate *p*-Value	Hazard Ratio (95% CI)
Sex	M	103	27	73 (4)	0.44		
	F	59	19	68 (6)			
Median age (years)	<10	75	23	69 (5)	0.73		
	≥10	87	23	74 (5)			
Median LDH (IU/L)	<375	81	25	70 (5)	0.63		
	≥375	81	21	73 (5)			
St. Jude Staging System *	I + II	35	6	83 (6)	0.1	0.39	
	III + IV	127	40	69 (4)			
Ann Arbor Staging System °	1 + 2	55	12	78 (6	0.14	0.93	
	3 + 4	107	34	68 (5)			
Risk group ^	LR + SR	62	15	77 (5)	0.31		
	HR	100	31	68 (5)			
Age-adjusted IPI (53 mv)	0–2	84	21	76 (5)	0.48		
	3	25	8	67 (10)			
B symptoms (2 mv)	No	64	17	73 (6)	0.61		
	Yes	96	28	71 (5)			
Mediastinum involvement	No	84	20	77 (5)	0.13	0.26	
	Yes	78	26	65 (5)			
Lung involvement	No	129	36	73 (4)	0.45		
	Yes	33	10	67 (9)			
Liver involvement	No	143	41	72 (4)	0.93		
	Yes	19	5	72 (11)			
Spleen involvement	No	133	39	71 (4)	0.8		
	Yes	29	7	74 (8)			
Skin lesions	No	119	30	75 (4)	0.21		
	Yes	43	16	63 (7)			
Soft tissue mass	No	132	42	68 (4)	0.11	0.28	
	Yes	16	2	88 (8)			
ENT involvement (4 mv)	No	158	44	72 (4)	-		
	Yes	0	0	-			
Peripheral LN involvement	No	20	2	90 (7)	0.06	0.11	
	Yes	142	44	69 (4)			
Abdominal LN involvement	No	90	25	72 (5)	0.86		
	Yes	72	21	71 (5)			
Visceral (lung, liver, spleen) involvement	No	111	31	73 (4)	0.55		
	Yes	51	15	69 (7)			
Kidney + pancreas lesions	No	153	44	71 (4)	0.69		
	Yes	9	2	78 (14)			
Bone lesions	No	128	39	70 (4)	0.23		
	Yes	34	7	79 (7)			
Bone marrow involvement	No	152	41	73 (4)	0.14	0.74	
	Yes	10	5	50 (16)			
ALK immuno-staining	Neg	0	0	-	-		
	Pos	162	46	72 (4)			
CD3 immunostaining (22 mv)	Neg	83	31	72 (4)	0.08	0.25	
	Pos	17	13	55 (9)			
Histological subtype SC/LH (8 mv)	No	95	19	81 (4)	0.0006	0.009	2.4 (1.23–4.66)
	Yes	59	27	53 (7)			
MDD	Neg	75	12	83 (4)	0.001	0.038	2.15 (1.04–4.64)
	Pos	87	34	62 (5)			

Events: progression or relapse; PFS: progression free survival; SE: standard error; M: male; F: female; LDH: lactate dehydrogenase; LR: low-risk; SR: standard-risk; HR: high-risk; IPI: International Prognostic Index; mv: missing value; ENT: ears nose throat; LN: lymph nodes; ALK: anaplastic lymphoma kinase; SC/LH: small cell or lymphohistiocytic; Neg: negative; Pos: positive; MDD: minimal disseminated disease; * Patients’ number according to St. Jude Stage System: 11 (stage I), 24 (stage II), 115 (stage III), 12 (stage IV); ° Patients’ number according to Ann Arbor Stage System: 13 (stage 1), 42 (stage 2), 51 (stage 3), 56 (stage 4); ^ Patients’ number according to LR and SR Risk Group: 3 (LR), 59 (SR).

## Supplementary Materials

The following are available online at https://www.mdpi.com/2072-6694/12/10/2747/s1, Figure S1: The 10-year OS analysis according to bp risk groups, Table S1: Therapy courses. Table S2: Univariate and multivariate analysis of relapse risk for 232 patients with available data for age-adjusted International Prognostic Index score and CD3 immunostaining.

## Appendix A

### Appendix A.1. Analysis of the Statistical Associations Among Clinical and Pathological Prognostic Factors for the 420 Eligible Patients

Mediastinal involvement, peripheral lymph nodes and SC/LH pattern showed a significant association with the risk of disease progression or relapse when assessed by univariate analysis.

Compared to patients without mediastinal involvement (227 patients), patients with mediastinal disease showed significantly higher levels of LDH above the median value (107/227 cases, 47% vs. 114/193 cases, 59%; *p* = 0.015), more often B-symptoms (90/227 cases, 40% vs. 140/188 cases, 75%; *p* < 0.0001), involvement of peripheral lymph nodes (192/227 cases, 85% vs. 180/193 cases, 93%; *p* = 0.005), visceral involvement (lung, liver or spleen, 40/227 cases, 18% vs. 96/193 cases, 50%; *p* < 0.0001) and BM disease (14/227 cases, 6% vs. 27/193 cases, 14%; *p* = 0.02).

Patients with peripheral lymph node involvement displayed HR characteristics (22/48 cases, 46% vs. 236/372 cases, 63%; *p* = 0.02), but were less often associated with soft tissue lesions (15/45, 33%, vs. 22/348, 6%; *p* < 0.0001) or bone involvement (19/48, 40% vs. 48/372, 13%, *p* < 0.0001), compared to patients without peripheral lymph node involvement.

The SC/LH pattern was significantly associated with mediastinal involvement (114/275 cases, 42% vs. 65/121, 54%; *p* = 0.02) and skin lesions (58/275, 21% vs. 46/121, 38%, *p* = 0.0004). In addition, the SC/LH pattern was associated with a higher LDH value (131/275 cases, 48% vs. 74/121 cases, 61%; *p* = 0.01), HR group (150/275 cases, 55% vs. 92/121 cases, 76%; *p* < 0.0001) and an absence of liver involvement (31/275 cases, 11% vs. 27/121 cases, 22%; *p* = 0.004) or visceral involvement (80/275 cases, 29% vs. 50/121 cases, 41%; *p* = 0.02).

### Appendix A.2. Analysis of the Statistical Associations among Clinical, Pathological and Biological Prognostic Factors for the 162 Patients with MDD Evaluation

By univariate analysis, only the SC/LH pattern and MDD-positivity significantly associated with a higher risk of disease progression or relapse. As for the whole group of patients, SC/LH pattern was associated with mediastinal involvement (38/95 cases, 40% vs. 35/59 cases, 59%; *p* = 0.02) and with HR characteristics (51/95 cases, 54% vs. 44/59 cases, 75%; *p* = 0.009). MDD was significantly associated with B-symptoms (31/75 cases, 41% vs. 65/85 cases, 76%; *p* < 0.0001), mediastinal involvement (29/75 cases, 39% vs. 49/87 cases, 56%; *p* = 0.03) and visceral involvement (17/75 cases, 23% vs. 34/87 cases, 39%; *p* = 0.03). No association between MDD and skin involvement was detected (*p* = 0.08). Consequently, MDD positivity strongly associated with the clinical HR group (37/75 cases, 49% vs. 63/87, 72%; *p* = 0.002) and with an increased LDH level (32/75 cases, 43% vs. 53/87, 61%; *p* = 0.02). In addition, MDD positivity was related to morphological BM involvement (1/75 cases, 1% vs. 9/87, 10%; *p* = 0.02). By contrast, we did not observe any relationship between MDD positivity and SC/LH pattern (24/69 cases, 35% vs. 35/85, 41%; *p* = 0.42).
