# Supplementary material for: Prognostic Factors in Childhood Anaplastic Large Cell Lymphoma: Long Term Results of the International ALCL99 Trial
Source: Cancers (Basel). 2020 Sep 24;12(10):2747. doi: 10.3390/cancers12102747 (PMC7598675; doi:10.3390/cancers12102747)
Supplement: Supplementary file 1 [file cancers-12-02747-s001.pdf]

cancers

Prognostic Factors in Childhood Anaplastic Large Cell Lymphoma: Long Term Results of the International ALCL99 Trial

Lara Mussolin, Marié-Cecilé Le Deley, Elisa Carraro, Christine Damm-Welk, Andishe Attarbaschi, Denise Williams, Amos Burke, Keizo Horibe, Atsuko Nakazawa, Grazyna Wrobel, Georg Mann, Monika Csóka, Anne Uyttebroeck, Rafael Fernández-Delgado Cerdá, Auke Beishuizen, Karin Mellgren, Birgit Burkhardt, Wolfram Klapper, Suzanne D. Turner, Emanuele S.G. D'Amore, Laurence Lamant, Alfred Reiter, Wilhelm Woessmann, Laurence Brugières, Marta Pillon and on behalf of the European Inter-Group for Childhood Non-Hodgkin lymphoma (EICNHL)

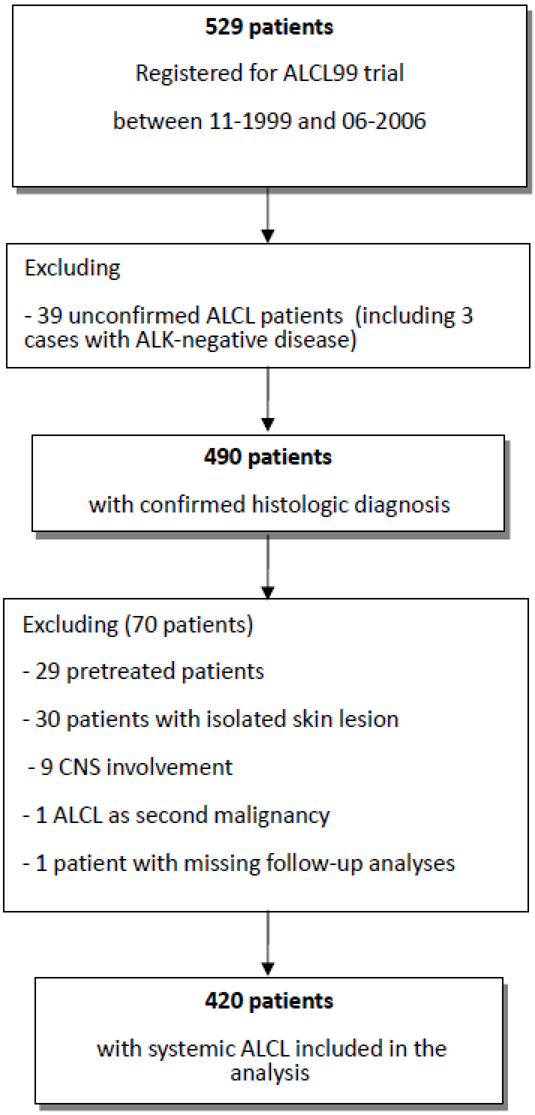

Figure S1. Flow chart of ALCL patients included in the analysis.

Table S1. Therapy courses.

Drug	Dose	Days				
		1	2	3	4	5
	PREPHASE					
Dexamethasone orally/IV [a]	5 mg/sqm/day	×	×			
	10 mg/sqm/day			×	×	×
Cyclophosphamide IV (1 h)	200 mg/sqm/day	×	×			
MTX + ARA-C + HSCH IT	12 mg + 30 mg + 10 mg [b]	×				
	COURSE A [c]					
Dexamethasone orally/IV [a]	10 mg/sqm/day	×	×	×	×	×
MTX IV	1st Random [d]: MTX1 (1 g/sqm over 24 h) or MTX3 (3 g/sqm over 3 h)	×				
Ifosfamide IV (1 h)	800 mg/sqm/day	×	×	×	×	×
ARA-C IV (1 h)	150 mg/sqm every 12 h				×-×	×-×
Etoposide IV (2 h)	100 mg/sqm/day				×	×
VBL IV (1 h)	2nd Random [e]: VBL (6 mg/sqm/day) or no VBL	×				
MTX + ARA-C + HSCH IT [f]	12 mg + 30 mg + 10 mg [b]	×				
	COURSE B [c]					
Dexamethasone orally/IV [a]	10 mg/sqm/day	×	×	×	×	×
MTX IV (1 h)	1st Random [d]: MTX1 (1 g/sqm over 24 h) or MTX3 (3 g/sqm over 3 h)	×				
Cyclophosphamide IV (1 h)	200 mg/sqm/day	×	×	×	×	×
Doxorubicin IV (1 h)	25 mg/sqm/day				×	×
VBL IV (1 h)	2nd Random [e]: VBL (6 mg/sqm/day) or no VBL	×				
MTX + ARA-C + HSCH IT [f]	12 mg + 30 mg + 10 mg [b]	×				

MTX = methotrexate; ARA-C = cytarabine; HSCT = hydrocortisone; h = hours; IT = intrathecal; IV = intravenously; VBL: vinblastine; [a] Subdivided in 2 or 3 doses; [b] Doses of IT chemotherapy were age-adjusted for children <3 years; [c] Low-risk patients received the Prephase, followed by courses A1, B1 and A2; [d] First randomisation occurred before course A1 for standard risk and high risk patients. Arm MTX1 included MTX 1 g/sqm in a 24-h infusion with triple IT injections at day 1 and leucovorin rescue (15 mg/sqm) at 42, 48 and 54 h (Arm 1: A1, B1, A2, B2, A3, B3). Arm MTX3 included MTX 3 g/sqm in a 3-h infusion with no IT injection and leucovorin rescue (15 mg/sqm every 6 h) starting at 24-h and ending when the MTX level was ≤0.15 m/L (Arm 3: AM1, BM1, AM2, BM2, AM3, BM3); [e] Second randomisation: additionally, high-risk patients could be entered into the 2nd randomized trial before the first course B (VBL trial), which randomly assigned patients to receive or not receive a VBL injection (6 mg/sqm) during the five latter courses (Arm 2 with MTX 1 g/sqm: BV1, AV2, BV2, AV3, BV3. Arm 4 with MTX 3 g/sqm: BMV1, AMV2, BMV2, AMV3, BMV3) and then weekly for a total treatment duration of 1 year. [f] IT only in Arm 1 and Arm 2, not in Arm 3 and Arm 4.

Table S2. Univariate and multivariate analysis of relapse risk for 232 patients with available data for age-adjusted International Prognostic Index score and CD3 immunostaining.

Patient characteristics		# Patients	# Events	10-y PFS % (SE %)	Univariate *p*-Value	Multivariate *p*-Value	Hazard Ratio (95% CI)
St. Jude Stage System *	I + II	109	28	74 (4)	0.17	0.32	
	III + IV	311	97	69 (3)			
Ann Arbour Stage System °	1 + 2	166	43	74 (4)	0.1	0.06	
	3 + 4	254	82	68 (3)			
Risk group ^	LR + SR	162	42	75 (4)	0.11	0.07	
	HR	258	83	63 (3)			
Age-adjusted IPI (91 mv)	0–2	263	68	74 (3)	0.02	0.99	
	3	66	26	59 (6)			
B symptoms (5 mv)	No	185	47	74 (3)	0.08	0.11	
	Yes	230	74	68 (3)			
Mediastinum involvement	No	227	58	75 (3)	0.03	0.34	
	Yes	193	67	65 (4)			
Peripheral LN involvement	No	48	7	88 (5)	0.02	0.06	
	Yes	372	118	68 (2)			

CD3 immunostaining (98 mv)	Neg	268	73	74 (3)	0.04	0.67	
	Pos	54	23	56 (7)			
Histological subtype SC/LH (24 mv)	No	275	61	79 (2)	<0.0001	<0.0001	2.74 (1.63–4.59)
	Yes	121	58	50 (5)			

° In the table only parameters with $p < 0.2$ determined by univariate analysis that were entered into the multivariate model are reported; * reference category. CI: confidence interval; LR: low-risk; SR: standard-risk; HR: high-risk; LN: lymph nodes; IPI: International Prognostic Index; Neg: negative; Pos: positive; SC/LH: Small Cell or Lymphohistiocytic. ^ Patients' number according to LR and SR Risk group: 6 (LR), 156 (SR).

© 2020 by the authors. Licensee MDPI, Basel, Switzerland. This article is an open access article distributed under the terms and conditions of the Creative Commons Attribution (CC BY) license (http://creativecommons.org/licenses/by/4.0/).
